# MINDY1 promotes breast cancer cell proliferation by stabilizing estrogen receptor α

**DOI:** 10.1038/s41419-021-04244-z

**Published:** 2021-10-13

**Authors:** Jianing Tang, Yongwen Luo, Guo Long, Ledu Zhou

**Affiliations:** 1 Department of Liver Surgery, Xiangya Hospital, Central South University, Changsha, China; 2 Department of Urology, Zhongnan Hospital of Wuhan University, Wuhan, China

**Keywords:** Cancer, Ubiquitylation

## Abstract

Breast cancer is the most commonly diagnosed malignant tumor among females. Estrogen receptor α (ERα) is initially expressed in 70% of breast cancers and is a well-known target of endocrine therapy for ERα-positive breast cancer. In the present study, we identified MINDY1, a member belongs to the motif interacting with Ubcontaining novel DUB family (MINDY), as a potential deubiquitylase of ERα in breast cancer. There was a positive correlation between ERα and MINDY1 protein levels in human breast cancer tissues. We found that high expression of MINDY1 was associated with poor prognosis. MINDY1 interacted with ERα, thereby mediating the deubiquitination of ERα and increased its stability in a deubiquitylation activity-dependent manner. MINDY1 depletion significantly decreased the ERα protein level and ERα signaling activity in breast cancer cells. Specifically, MINDY1 associated with the N-terminal of ERα via its catalytic domain, thus inhibiting K48-specific poly-ubiquitination process on ERα protein. In addition, MINDY1 depletion led to growth inhibition and cell cycle arrest of ERα-positive breast cancer cells. Finally, overexpression of ERα could rescue the MINDY1 depletion-induced growth inhibition both in vitro and in vivo, suggesting that MINDY1 promotes breast carcinogenesis through increasing ERα stability. Overall, our study proposed a novel post-translational mechanism of ERα in supporting breast cancer progression. Targeting the MINDY1 may prove to be a promising strategy for patients with ERα-positive breast cancer.

## Introduction

Breast cancer is a major health burden in female around the world, which causes the most frequent women cancer prevalence and results in the second leading cause of cancer-related death in women worldwide [1]. Breast cancer has been divided into at least three subtypes (Luminal, HER2-enriched, and triple-negative) based on the expression of estrogen receptor (ER), progesterone receptor (PR), and human epidermal growth factor receptor 2 (HER-2). These subtypes exhibit different histopathological features and treatment sensitivities [2].

Estrogen receptor alpha (ERα) is overexpressed in approximately 70% of all breast cancer cases. ERα is a well-known biomarker and is considered as one of the most successful molecular targets for endocrine therapy [3, 4]. ERα-positive breast cancers could be controlled by the modulators of ERα, such as tamoxifen [5]. However, it is common to develop acquired resistance of tamoxifen, making it an important clinical issue in breast cancer therapy [6]. ERα is ligand-activated transcription factors composed of three functional domains for hormone binding, DNA binding, and transcriptional activation. The ligand-binding domain (LBD) is recognized by the 17 Beta Estradiol Hormone (E2). Transactivation domains AF-1 and AF-2 cooperate in transactivation of ERα. And the DNA-binding domain (DBD) recognizes the estrogen-responsive element on the DNA [7, 8]. ERα plays a central role in the signaling transduction pathway of breast cancer cells, and upregulation of ERα is associated with the initiation and progression of breast cancer [9, 10]. ERα could increase the expression level of oncogenic proteins, including cyclin D1 and c-myc, while it inhibits the level of cell cycle inhibitors, including P21 [11]. Overexpression of ERα promotes breast cancer cell growth and the activity of ERα is essential for cell cycle progression, which could accelerate the G1-S phase transition [12]. Since ERα and its signaling pathways have crucial roles in the initiation and development of breast cancer, anti-estrogen therapy and targeting ERα signaling are important parts of the treatment for patients with ERα-positive breast cancer. However, endocrine resistance is the major clinical problem. There are two types of endocrine resistance: primary endocrine resistance and secondary resistance. The primary endocrine resistance is defined as relapse during the first 2 years of adjuvant endocrine therapy or progressive disease within the first 6 months of first-line endocrine therapy for metastatic breast cancer. The secondary resistance is defined as relapse while on adjuvant endocrine therapy but after the first 2 years of treatment, relapse within 12 months of completing adjuvant endocrine therapy, or progressive disease six or more months after starting endocrine therapy for metastatic breast cancer [13]. Endocrine resistance is inevitable after prolonged exposure to endocrine therapies [14]. The effectiveness of endocrine therapies is largely limited due to primary and secondary endocrine resistance [15]. Thus, a deeper understanding of the dysregulation of ERα signaling will facilitate the development of new strategies for the treatment of patients with breast cancer.

The ERα protein stability and turnover were shown to account for hyper-activation of ERα and endocrine resistance in breast cancer [16]. Previous studies have indicated that ubiquitin-proteasome system (UPS) is involved in the regulation of ERα stability. The E3-ubiquitin ligases including MDM2, BRCA1, SKP2, BARD1, CHIP, and E6AP can induce the 26S proteasome-mediated degradation of ERα by the increase the poly-ubiquitin to ERα lysine residues [17–22]. Deubiquitinating enzymes (DUB) are also involved in the regulation of ERα in breast cancer. A previous study demonstrated that USP7 was a potential deubiquitylase of ERα and promoted breast cancer progression [23]. However, to explore exact mechanisms underlying ERα dysfunction is still in need of further investigation.

In the present study, we observed that the expression of MINDY1 was positively correlated with ERα protein level in clinical breast tissues. MINDY1 may function as a deubiquitinase responsible for ERα deubiquitination and stabilization. Further investigations revealed that MINDY1 promoted the proliferation and migration of breast cancer cells through ERα.

## Materials and methods

### cBioPortal analysis

We used cBioPortal (http://www.cbioportal.org) to inquire into the gene alteration status of MINDYs in breast cancer. The cBioPortal for Cancer Genomics provides online resources for the exploration, visualization, and analysis of multidimensional cancer genomics data. The OncoPrint schematic was constructed in cBioPortal (TCGA dataset) to directly reflect all types of alterations such as amplification, deep deletion of the MINDY genes from 1084 breast cancer patients.

### Cell culture

Human embryonic kidney HEK293 cell line and ERα-positive human breast cancer cell lines MCF-7, T47D were purchased from American Type Culture Collection (ATCC). T47D cells were cultured in RPMI-1640 (HyClone, USA) supplemented with 10% fetal bovine serum (FBS, HyClone). MCF-7 and HEK293 cells were culture with Dulbecco’s Modified Eagle’s Medium (DMEM) that contains 4 mM L-glutamine and 4,5 g/L glucose (HyClone, USA) supplemented with 10% FBS. All cells were cultured at 37 °C in an atmosphere of 5% CO_2_.

### Plasmids and RNA inference

Wild type (WT) MINDY1 and its deletion mutant plasmids were acquired from Hanbio Biotechnology Co., Ltd. (Shanghai, China). Small interfering RNAs targeting MINDY1(siG000055793A-1-5, siG000055793B-1-5) were obtained from Ruibo Biotechnology Co., Ltd. (Guangzhou, China). The ERα full- and its deletion constructs were gifted from Dr. Ting Zhuang and were described in our previous study [24]. The HA-K6, -K11, -K27, -K29, -K33, -K48, -K63, and Ub plasmids were described in our previous study [25]. Plasmids and small interfering RNAs were transfected into cells using Lipofectamine 2000 (Invitrogen, Carlsbad, CA, USA) according to the manufacturer’s instructions.

### Cell proliferation analysis

The cell proliferation analysis was performed using Cell Counting Kit-8 (CCK8) assay and EdU incorporation assay as we previously described [25]. All experiments were independently repeated three times with three replicates.

### Cell migration analysis

For cell migration analysis, the wound-healing assay was performed. MCF-7 and T47D cells were seeded in 6-well plates and allowed to reach full confluent. Cells were scraped with a 200 μl pipette tip and washed with PBS three times. Each well was subsequently filled with fresh medium containing 1% FBS. The migration distances of the cells were measured at the indicated time points. Each experiment was performed at least three times.

### Animal experiments

For xenograft tumor model, female BALB/c nude mice aged 4 weeks were purchased from Vital River (Beijing, China) and animal protocols were approved by the Ethics Committee at Xiangya Hospital of Central South University. The nude mice were implanted with 0.72 mg/90-day-release-17β-estradiol pellets for 1 week. Animals were randomly divided into different groups (*n* = 8 per group). Stably-transfected MCF-7 cells were suspended in PBS (2 × 10^6^ cells/100 μl) and injected into the mammary fat pad. The tumor volume was measured every 5 days until the end of the experiment.

### Co-immunoprecipitation assay

Co-immunoprecipitation assay was performed as we previously described [25]. Briefly, cells were lysed with NP-40 lysis buffer and immunoprecipitated with the indicated antibody at 4 °C overnight. The immunocomplexes were pulled down using protein A/G PLUS-Agarose beads (Santa Cruz) and separated by western blotting.

### GST pulldown assays

GST pulldown assays were performed as we previously described [25]. Briefly, glutathione agarose beads were incubated with purified proteins overnight. The beads were then washed with GST binding buffer. The bound proteins were separated by western blot.

### In vivo deubiquitination assay

In vivo deubiquitination assay was performed as we previously described [25].

### Western blot analysis

Proteins were extracted from cultured cells using RIPA extraction reagent (Beyotime, China) supplemented with protease inhibitors (Sigma-Aldrich, USA), followed by immunoblotting with the corresponding antibodies: ERα (CST, 8644), Myc (Proteintech, 60003-2-Ig), HA (Proteintech, 51064-2-AP), MINDY1 (Invitrogen, PA5-55825), and GAPDH (Proteintech, 60004-1-Ig) antibodies.

### Statistical analysis

Prism 7.0 (GraphPad, USA) was used for statistical analysis. Cumulative survival rates were estimated by using the Kaplan–Meier method and compared with the log-rank test. Student’s *t* test and one-way ANOVA were used to compare two and more groups respectively. *P* < 0.05 was considered to indicate statistical significance; all tests were two-tailed.

## Results

### MINDY1 depletion inhibits ERα signaling pathway activity

**Fig. 1:**
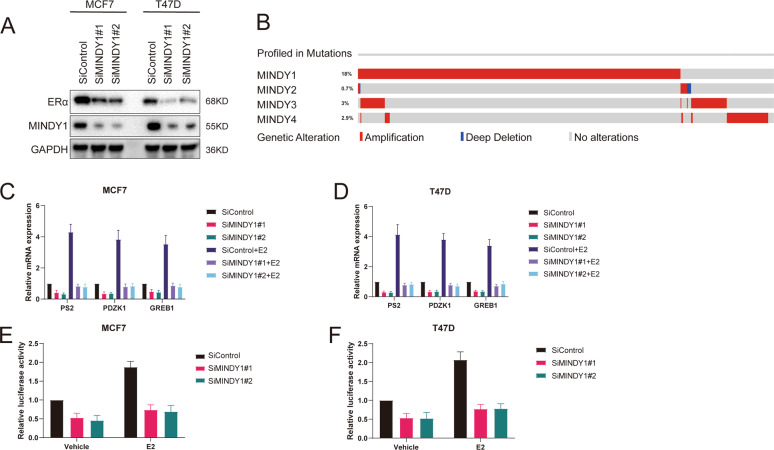
MINDY1 depletion decreases ERα signaling activity in breast cancer cells. **A** MINDY1 depletion decreased ERα protein level. **B** Genetic alternations of MINDYs in breast cancer. MINDY1 was amplificated in 18% of breast cancer patients. The OncoPrint schematic was constructed in cBioPortal (TCGA dataset) to directly reflect all types of alterations such as amplification, deep deletion of the MINDY genes from 1084 breast cancer patients. **C**, **D** MINDY1 depletion decreased ERα target genes in the absence or presence of estrogen. Breast cancer cells were transfected with si MINDY1 or siControl. After 48 h, cells were treated with either ethanol or 10 nM estrogen for 6 h. Total RNA was prepared and the expression of the endogenous ERα target genes, PS2, GREB1, and PDZK1 were determined by qRT-PCR. **E**, **F** MINDY1 depletion affected ERE-luciferase activity. Breast cancer cells were transfected with siMINDY1 or siControl together with ERE luciferase reporter plasmid. Cells were treated with 10 nM estrogen or vehicle. Luciferase activity was measured 48 h after transfection. The experiment was independently repeated three times with three replicates. *P value < 0.05; **P value < 0.01; ***P value < 0.001.

To identify the potential roles of MINDYs responsible for ERα deubiquitination and stabilization in ERα-positive breast cancer, We transfected four nonoverlapping siRNA mixtures specific for each MINDYs into MCF-7 cells. We observed that MINDY1 depletion significantly decreased ERα (Fig. S1A). We used two non-overlapping siRNAs targeting MINDY1 to further validate the function of MINDY1 in regulating ERα, as shown in Fig. 1A and Fig. S1B, MINDY1 depletion significantly decreased the ERα protein levels without influence on the expression of ERα mRNA. Besides, MINDY1 depletion decreased ERα protein level in both estrogen and vehicle conditions (Fig. S1C). Genomic analysis of all the MINDYs in human breast cancer samples revealed MINDY1 amplification was observed in 18% of cases (Fig. 1B). MINDY1 depletion significantly reduced the expression of endogenous ERα target genes such as PS2, GREB1, and PDZK1 in the presence or absence of estrogen (Fig. 1C, D). The ERα-luciferase reporter gene activity was measured to determine whether MINDY1 depletion affected the transcriptional activity of ERα. It was observed that MINDY1 depletion inhibited the activity of ERα-luciferase reporter gene both in the presence or absence of estrogen (Fig. 1E, F). Consistently, Overexpression of MINDY1 significantly enhanced ERα transcriptional activity (Fig. S1D). All these results demonstrated that MINDY1 was a regulator of the ERα signaling pathway.

### MINDY1 is associated with ERα protein levels in human breast cancer samples and poor prognosis

**Fig. 2:**
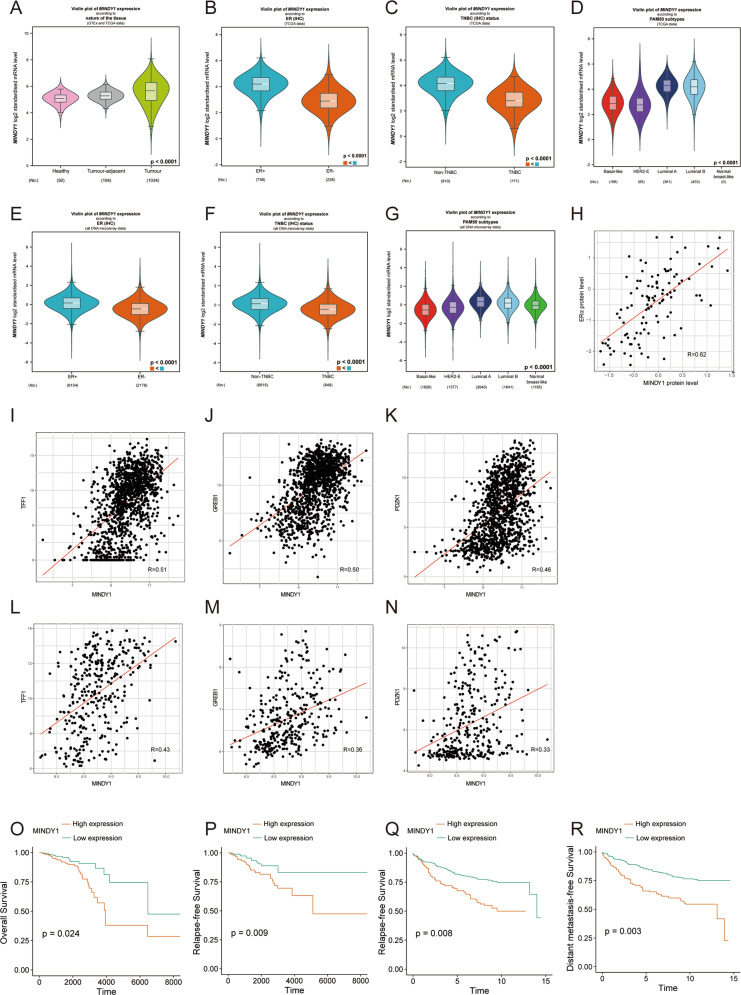
MINDY1 is overexpressed in breast cancer and correlates with poor prognosis. **A**–**G** Expression of MINDY1 in breast cancer. **A**–**D** Patients were extracted from TCGA BRCA dataset. **E**–**G** Patients were extracted from microarrays datasets. All data are available at bc-GenExMiner v4.5 (http://bcgenex.centregauducheau.fr/BC-GEM/GEM-Accueil.php?js=1). The significance of differences was calculated using one-way ANOVA test. **H** Correlations between MINDY1 and ERα protein levels in CPTAC. **I**–**K** Correlations between MINDY1 and ERα target genes in TCGA. **L**–**N** Correlations between MINDY1 and ERα target genes in GSR6532. **O**, **P** MINDY1 is associated with poor overall survival and relapse-free survival of breast cancer patients in TCGA (*n* = 1201). Cox proportion hazards model was used to understand the significance between two groups. **Q**–**R** MINDY1 was associated with poor relapse-free survival and distant metastases-free survival of ERα-positive breast cancer patients in GSE6532 (*n* = 327). Cox proportion hazards model was used to clarify the significance between two groups. *P value < 0.05; **P value < 0.01; ***P valu*e* < 0.001.

**Fig. 3:**
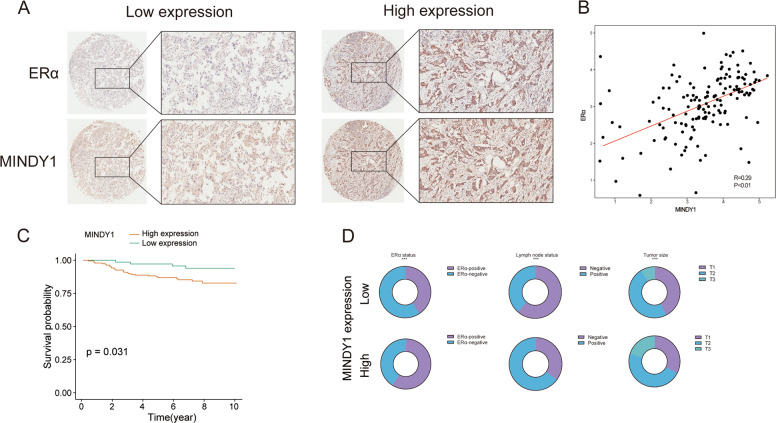
MINDY1 correlates with ERα protein levels and poor prognosis in human breast cancer samples. Tissue microarray was obtained from Shanghai Biochip Company Co., Ltd, Shanghai, China. The tissue microarray contained 140 breast cancer specimens. **A** The typical staining of ERα and MINDY1 in breast cancer specimens. **B** ERα positively correlated with MINDY1 in breast cancer samples (Pearson correlation). **C** High expression of MINDY1 was associated with poor prognosis. Cox proportion hazards model was used to understand the significance between two groups. **D** MINDY1 expression was associated with the ERα status, lymph node status and tumor size. The characteristics were compared between MINDY1 low-/high- groups using chi-square or Fisher’s exact tests. *P value < 0.05; **P value < 0.01; ***P value < 0.001.

We analyzed MINDY1 expression in breast cancers using bc-GenExMiner v4.5(http://bcgenex.centregauducheau.fr/BC-GEM/GEM-Accueil.php?js=1), which offers the possibility to explore gene-expression of genes of interest in breast cancer. As shown in Fig. 2, MINDY1 was highly expressed in breast cancer samples, especially in the luminal A subtype (Fig. 2A–G). We observed a positive correlation between MINDY1 and ERα protein levels based on the analysis of 105 TCGA breast cancer samples from the Clinical Proteomic Tumor Analysis Consortium (https://cptac-data-portal.georgetown.edu/cptacPublic/) (Fig. 2H). Consistently, MINDY1 was positively correlated with PS2, PDZK1, and GREB1 expression based on the analysis of TCGA database and GSE6532 (Fig. 2I–N). It was found that MINDY1 expression was a poor prognostic factor for breast cancer patients bas(Fig. 2O, P). As MINDY1 was upregulated in ERα-positive breast cancer patients and associated with the ERα protein level, we then analyzed its prognostic value in ERα-positive breast cancer from GES6532, and observed that high expression of MINDY1 was associated with poor prognosis of patients with ERα-positive breast cancer (Fig. 2Q, R). We performed immunohistochemistry (IHC) analysis of two tissue microarrays (TMA) to explore the correlations between MINDY1 and ERα staining (Fig. 3A, B). It was found that ERα staining was positively correlated with MINDY1, suggesting the potential regulatory network between ERα and MINDY1. And high expression of MINDY1 was correlated with poor clinical outcomes (Fig. 3C). Further analysis demonstrated that MINDY1 expression was correlated the ERα status, the lymph node metastasis status and tumor size (Fig. 3D).

### MINDY1 interacts with ERα

**Fig. 4:**
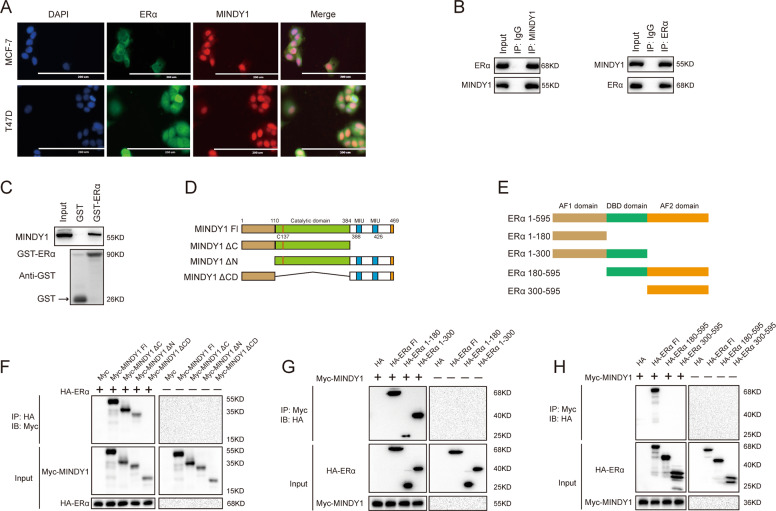
MINDY1 associates with ERα. **A** An immunofluorescence assay demonstrated that MINDY1 and ERα at least partially colocalized in MCF7 and T47D cells. **B** Co-IP assay revealed an association between endogenous MINDY1 and ERα in MCF-7 cells. MCF-7 cells were harvested with RIPA lysis buffer. Co-IP was performed using antibody as indicated. **C** Purified His-MINDY1 was incubated with GST- ERα or GST protein. The interacted MINDY1 was detected via western blot. **D** ER alpha domain structure and deletion mutants used in the study. **D**, **E** ERα and MINDY1 domain structure and deletion mutants used in the study. **F** The catalytic domain of MINDY1 interacted with ERα. HEK293 cells were transfected with 2 µg HA-ER alpha together with Myc-MINDY1 full length or mutants. After 24 h, cells were harvested with NP-40 lysis buffer. Co-IP was performed using HA antibody. The possible interacted MINDY1 domains were detected by Myc antibody. **G**, **H** MINDY1 interacted with ERα through its AF1 domain. HEK293 cells were transfected with 2 µg Myc-MINDY1 together with HA-ERα full length or mutants. After 24 h, cells were harvested with NP-40 lysis buffer. Co-IP was performed using Myc antibody. The possible interacted ERα domains were detected by HA antibody. The experiment was independently repeated three times with three replicates.

An immunofluorescence assay showed that ERα and MINDY1 localized both in the nucleus and cytosol of breast cancer cells (Fig. 4A). Endogenous MINDY1 and ERα from lysates of MCF-7 cells were co-immunoprecipitated, suggesting the interaction of MINDY1 and ERα in the physiological condition (Fig. 4B). Furthermore, GST-pull-down assay confirmed the direct interaction between MINDY1 and ERα (Fig. 4C). We generated the deletion mutants of MINDY1. And the co-IP experiments revealed that MINDY1 mutants which included catalytic (CA) domain were able to interact with ERα. Additionally, we found that the N-terminal of ERα which includes AF1 domain mediated the interaction with MINDY1 (Fig. 4D–H).

### MINDY1 deubiquitylates ERα

**Fig. 5:**
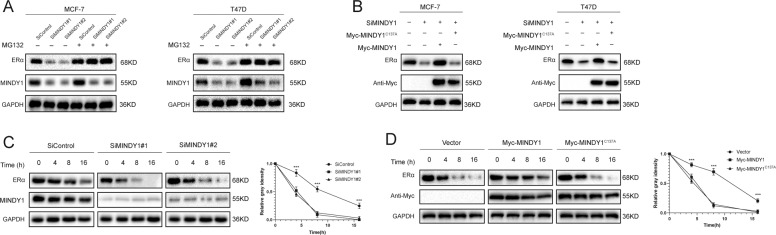
MINDY1 increases ERα stability. **A** In the presence of the proteasome inhibitor MG132, depletion of MINDY1 did not further decrease the ERα protein level. Breast cancer cells were transfected with siMINDY1 or siControl. After 48 h, cells were treated with 10 µM MG132/vehicle for 6 h, cell lysates were prepared for western blot analysis. **B** MCF-7 cells were transfected with MINDY1 (wild type or C137A) together with MINDY1 siRNA. The ERα level were measured. **C** MINDY1 depletion decreased ERα half-life in breast cancer cells. Breast cancer cells were transfected with siMINDY1 or siControl. After 48 h, cells were treated with 100 µM cycloheximide/vehicle for indicated times. Cell lysates were prepared for western blot analysis. **D** MINDY1^C137A^ did not increase ERα half-life in HEK293 cells. HEK293 cells were transfected with HA-ERα plasmid and Myc-tag, Myc-MINDY1 or Myc- MINDY1^C137A^ plasmids. After 24 h, cells were treated with 100 µM cycloheximide/vehicle for indicated times. Cell lysates were prepared for western blot analysis. The experiment was independently repeated three times with three replicates.

**Fig. 6:**
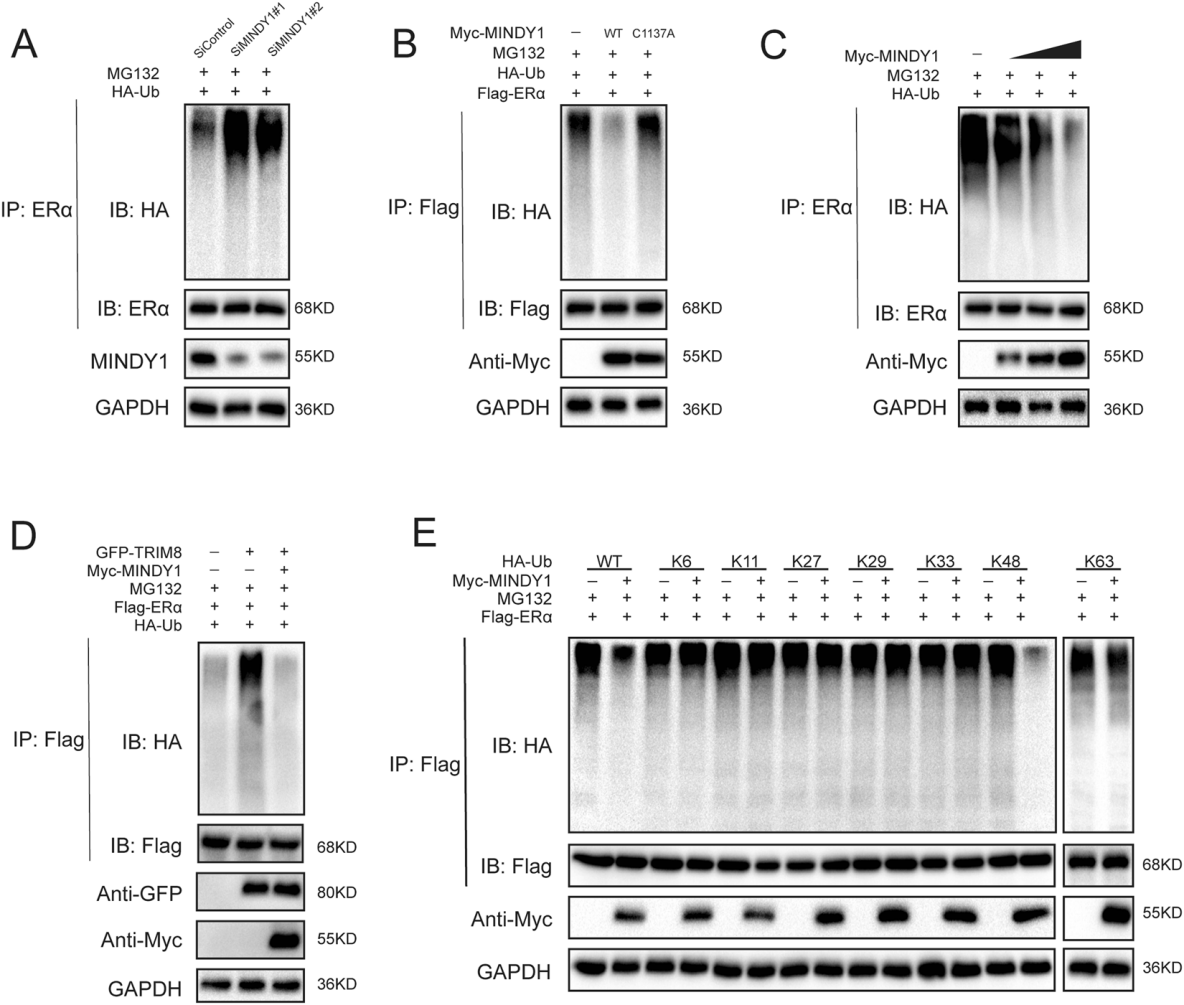
MINDY1 deubiquitylates ERα. **A** MCF-7 cells transfected with the indicated siRNA were treated with MG132 for 6 h before collection. ERα was immunoprecipitated with anti-ERα and immunoblotted with anti-HA. **B** Immunoblotting to detect the ubiquitination of ERα in HEK293 cells co-transfected with Flag- ERα, HA-Ubiquitin, and Myc-MINDY1 (wild type or C137A). **C** MINDY1 removed the ubiquitin chain of ERα in a dose-dependent manner. **D** ERα ubiquitylation was analyzed in cells transfected with E3 TRIM8 together with MINDY1 or not. **E** HA-WT, K6, K11, K27, K29, K33, K48, or K63 Ub was co-transfected with Flag-ERα and Myc- MINDY1 into HEK293 cells. After treatment with 10 μM MG132 for 6 h, cell lysates were subjected to ubiquitination assay and the ubiquitination level of ERα was detected by HA antibody. The experiment was independently repeated three times with three replicates. *P value < 0.05; **P value < 0.01; ***P value < *0*.001.

MINDY1 is a deubiquitinating enzyme that catalyzes removal of ubiquitin from its substrates. We hypothesized that ERα may be a substrate of MINDY1, and therefore we assessed whether MINDY1 regulated ERα deubiquitination. It was found that depletion of MINDY1 by siRNAs significantly decreased ERα protein level. The decrease of ERα was reversed by overexpression of the wild type, but not catalytically inactive mutant, MINDY1 or addition of the proteasome inhibitor MG132 (Fig. 5A, B). In order to prove that MINDY1 affected ERα stability, cells were treated with the protein synthesis inhibitor cycloheximide (CHX). In cells depleted of MINDY1, the half-life of ERα was significantly shortened (Fig. 5C). While the half-life of ERα was largely prolonged in cells overexpressing the wild type MINDY1, but not the catalytically inactive mutant MINDY1^C137A^ (Fig. 5D). We went on to test the possibility that MINDY1 deubiquitylates ERα. As show in Fig. 6A, MINDY1 depletion significantly increased ERα ubiquitylation in MCF-7 cells. Conversely, ectopic expression of the wild type MINDY1, but not its inactive mutant MINDY1^C137A^, reduced ERα polyubiquitylation in cells both in vivo and in vitro (Fig. 6B, Fig. S2). We observed that MINDY1 reduced ERα ubiquitylation in MCF-7 cells in a dose-dependent manner (Fig. 6C). Furthermore, MINDY1 reduced the ubiquitylation of ERα induced by the E3 ligase TRIM8 (Fig. 6D) [26]. To further investigate which type of ubiquitin chain of ERα was influenced by MINDY1, we performed ubiquitination assay with a series of ubiquitin mutants. It was found that MINDY1 could only remove the K48-linked ubiquitin chain from ERα protein (Fig. 6E). Collectively, these results indicated MINDY1 was a specific DUB, which de-polyubiquitylated and stabilized ERα.

### MINDY1 regulates cell proliferation and migration through ERα

**Fig. 7:**
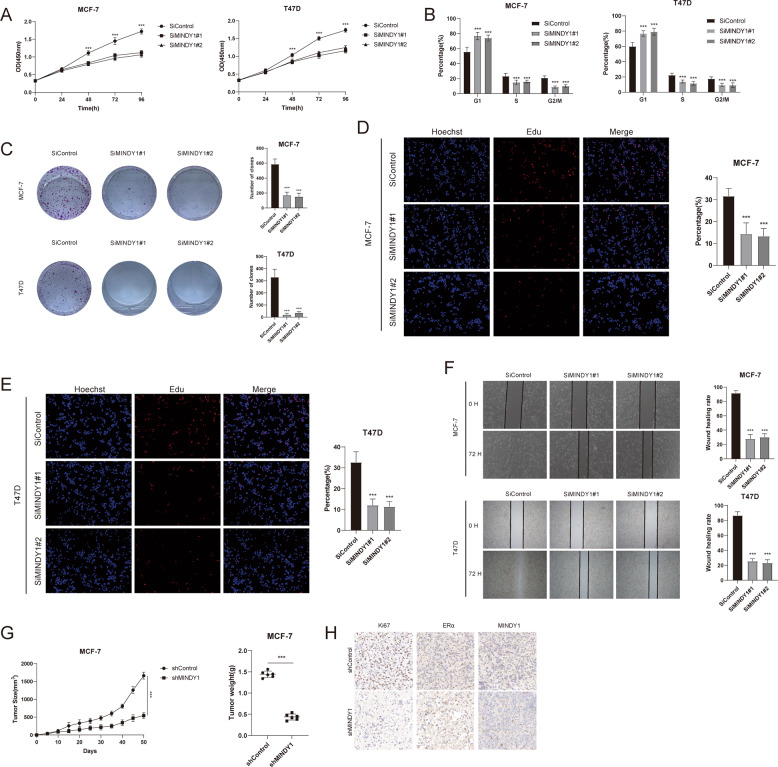
MINDY1 depletion inhibits ERα-positive breast cancer cell proliferation and migration. **A** MINDY1 depletion inhibited cell proliferation in breast cancer cells. **B** MINDY1 depletion induced G1 cell cycle arrest in breast cancer cells. **C** MINDY1 depletion decreased clone formation capability of breast cancer cells. **D**, **E** Representative images of EdU assay of breast cancer cells. **F** Wound-healing assay of breast cancer cells. MCF-7 and T47D cells were transfected with indicated 50 nM MINDY1 siRNA or 50 nM control siRNA. 24 h after transfection, cells were seeded into 6-well plates with 1% FBS with 100% confluence. A straight scratch was made on the cell layer with a yellow pipette tip. Quantification of wound closure was measured every 24 h, and the ERα protein level was measured at the endpoint. **G** MINDY1 depletion inhibits the cell proliferation in breast cancer cells in vivo. MCF-7 cells were stably transfected with lentivirus carrying a scrambled shRNA or MINDY1 shRNA. Female BALB/c nude mice were estrogen-supplemented by implantation of slow-release 17β-estradiol pellets (0.72 mg/90d release) 1 day before MCF-7 tumor cell injection into the mammary fat pad (2 × 10^6^ MCF-7 cells suspended in 100 μl Matrigel solution). MCF-7 tumor xenografts were measured every 5 days. **H** Representative images of immunohistochemical staining for Ki67, MINDY1, and ERα. The experiment was independently repeated three times with three replicates. *P value < 0.05; **P value < 0.01; ***P value < 0.001.

**Fig. 8:**
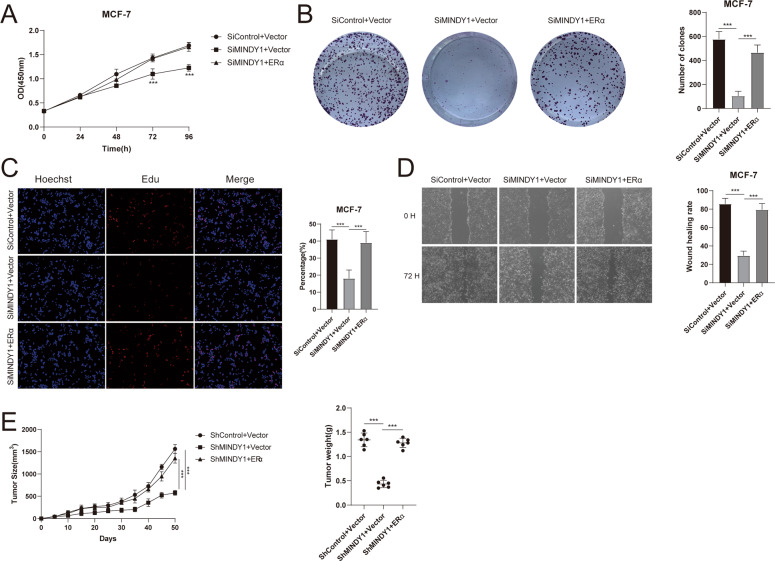
Increased ERα expression reverses the effect of MINDY1 depletion. **A** Cell proliferation assay of MCF-7. **B** Clone formation assay of MCF-7. **C** Representative images of EdU assay of breast cancer cells. **D** Wound-healing assay of MCF-7. **E** Overexpression of ERα in MINDY1-knockdown cells recovered tumor growth in vivo. The experiment was independently repeated three times with three replicates. *P value < 0.05; **P value < 0.01; ***P value < 0.001.

We further investigated the biological functions of MINDY1 in ERα-positive breast cancer cells. It was found that MINDY1 depletion inhibited cell proliferation in both vehicle and estradiol treated conditions (Fig. 7A and Fig. S3A). Depletion of MINDY1 induced G1 phases cell cycle arrest, indicating that MINDY1 was involved in the G1 to S transition of ERα-positive breast cancer cells (Fig. 7B). Subsequently, clone formation assays demonstrated that MINDY1 depletion significantly inhibited the clone formation ability of MCF-7 and T47D cells (Fig. 7C). EdU incorporation assay was further performed to measure the DNA synthesis. Our results indicated that MINDY1 depletion inhibited the DNA synthesis of MCF-7 and T47D cells (Fig. 7D, E). Furthermore, wound-healing assay revealed that depletion of MINDY1 significantly decreased the cell migration ability (Fig. 7F). Then, we used xenograft mice models to further investigated if MINDY1 promoted tumor growth in vivo. Xenograft tumor assay showed knockdown of MINDY1 markedly suppressed tumor growth (Fig. 7G, H). Tamoxifen is an antagonist of estrogen through binding to ERα and is applied to clinical therapy in patients with breast cancer. However, drug tolerance decreases the anti-tumor effects of tamoxifen. We explore if MINDY1 can improve the anti-tumor function of tamoxifen in ERα+ breast cancer cells. We test cell viability under MINDY1 siRNA or tamoxifen treatments. The results showed that the combination treatment of silencing MINDY1 expression and tamoxifen induced more obvious inhibitory effects and apoptosis in MCF-7 cells (Fig. S3B, C). To further identify MINDY1 promoted ERα-positive breast cancer cell proliferation and migration by increasing ERα stabilization, we ectopic expressed ERα in MINDY1 knockdown MCF-7 cells. Cell proliferation assays indicated that ectopic expression of ERα largely reversed the growth inhibition induced by MINDY1 depletion (Fig. 8A–C). Wound healing assay showed that overexpression of ERα could largely increase the ability of migration in MINDY1 knockdown cells (Fig. 8D). Xenograft mice models showed that the restoration of ERα expression in MINDY1 knockdown MCF-7 cells abolished the growth inhibition induced by MINDY1 depletion (Fig. 8E). Collectively, these results suggested that MINDY1 promoted breast cancer cell proliferation and migration, at least partially, via the stabilization of ERα.

## Discussion

Breast cancer is the most commonly diagnosed malignant tumor among females. And ER-positive breast cancers consist over 70% of breast cancers [5]. ERα was originally cloned from MCF-7 cell in 1985, which belongs to the nuclear receptor superfamily of transcriptional factors, [27]. Targeting ERα-signaling pathway is the main therapeutic strategy for the treatment of ERα-positive breast cancer patients because of its sensitivity and effectiveness [28]. However, over 30% of patients receiving endocrine therapy relapse with resistant disease, either through inherent resistance to treatment or the emergence of acquired endocrine resistance [29]. Several confirmed and hypothetical mechanisms influencing resistance to endocrine therapy have been identified. Several studies have demonstrated that FGFR1 amplifications play an important role in resistance to endocrine therapy. Overexpression of FGFR1 may contribute the poor prognosis of ERα-positive breast cancers and drive anchorage-independent proliferation and endocrine therapy resistance [30]. The mutations involved in ERα ligand-binding domain (LBD) and AF2 domain are unveiled in recent years as an important mechanism of acquired endocrine resistance [31]. Post-translational modifications of ERα, including phosphorylation, ubiquitination, and acetylation contribute the endocrine therapy resistance. P300 was reported to promote the ER signaling activity through acetylating the ERα at lysine residues within the ligand-binding domain of ERα [32]. The phosphorylation of ERα at certain sites can affect the ERα function in breast cancer cells. For example, the phosphorylation of ERα at Y537 site changes helix loop conformation and then increases the ligand binding/coactivator binding ability [33, 34]. Accumulating studies have indicated that the ubiquitin-proteasome system is tightly linked to ERα signaling activity. However, the DUBs responsible for ERα stabilization are less well understood. In the present study, we identified that MINDY1, which was highly expressed in ERα-positive breast cancer samples, was a novel post-translational modulator of ERα. MINDY1 interacted with ERα and increased its stabilization through removing the K48-linked ubiquitin from ERα. In addition, MINDY1 could promote the proliferation and migration of breast cancer cells by stabilizing ERα.

Ubiquitination is an important post-translational modification, which is a central component of the cellular protein-degradation machinery and essential for cellular homeostasis [35]. The major part of ubiquitination process is mediated by three enzymes: ubiquitin-activating enzyme (E1), a ubiquitin conjugating enzyme (E2) and a ubiquitin ligase (E3) [36]. It should be noted that the ubiquitination of cellular proteins is a reversible and dynamic process, constantly being ubiquitinated and deubiquitinated. This process is precisely and orchestrated determined by several E3 ubiquitin ligases and DUBs [37–39]. The E3 ubiquitin ligases selectively mediate the ubiquitin conjugation of substrates, while DUBs negatively regulate this process [40]. Accumulating evidence has confirmed that DUBs play an important role in cancer progression. The DUBs in the human genome can be categorized into six families: ubiquitin COOH-terminal hydrolases (UCH), ubiquitin-specific proteases (USP), the JAB1/MPN/MOV34 family (JAMM), Josephins, ovarian tumor proteases (OTU), and motif interacting with ubiquitin-containing novel DUB family (MINDY) [41]. MINDY1 was reported to contain MIU motifs with high selectivity for binding K48-linked polyUb, and DUB assays showed that full-length of MINDY1 had unique selectivity for cleaving K48-linked polyUb [41].

In this study, we first identified that MINDY1 as a novel post-translational modulator in the regulation of ERα ubiquitination and stability. It is found that ERα protein level and ERα signaling activity were significantly inhibited by MINDY1 depletion. We analyzed the public data available in bc-GenExMiner and found that MINDY1 was highly expressed in ERα-positive breast cancer samples. Based on the analysis of tissue microarrays and the Clinical Proteomic Tumor Analysis Consortium database, we observed an intimate correlation between MINDY1 expression and ERα protein level. Furthermore, survival analysis indicated that high expression of MINDY1 was associated with poor prognosis. We further investigated the underlying mechanism of MINDY1 in regulating ERα signaling. It was found that depletion of MINDY1 by siRNAs significantly decreased ERα protein level. Interestingly, the decrease of ERα could be reversed by overexpression of the wild type MINDY1, but not the catalytically inactive mutant, suggesting that MINDY1 modulated the stability of ERα through its catalytical activity. We treated cells with the CHX to prove if MINDY1 affected ERα stability. In cells depleted of MINDY1, the half-life of ERα was significantly shortened. While the half-life of ERα was largely prolonged in cells overexpressing the wild type MINDY1. We found that the decreased ERα expression induced by MINDY1 depletion could be largely recovered by addition of MG132, suggesting MINDY1 regulates ERα degradation through ubiquitin-proteasome system (UPS). We also identified that ERα and MINDY1 localized both in the nucleus and cytosol of breast cancer cells. The Co-IP experiment demonstrated that endogenous MINDY1 and ERα from lysates of MCF-7 cells were co-immunoprecipitated, indicating the interaction of MINDY1 and ERα in the physiological condition. GST-pull-down assay confirmed the direct interaction between MINDY1 and ERα. We further found that MINDY1 could only remove the K48-linked ubiquitin chain from ERα protein, thus inhibiting proteasome-mediated ERα degradation. Importantly, the catalytically inactive mutant of MINDY1 (C137A) lost the ability in modulating ERα, indicating that MINDY1 promoted ERα stability through its enzymatically active site. Our data further demonstrated that MINDY1 depletion reduced ERα-positive breast cancer growth both in vitro and in vivo. And the growth inhibition induced by MINDY1 depletion could be reversed by ectopic expression of ERα. These results demonstrated that MINDY1 promoted breast cancer proliferation and migration through increasing ERα stability.

## Conclusion

In our present study, we investigated the biological functions of MINDY1 in ERα-positive breast cancer cells, and we identified MINDY1 as a novel post-translational modulator in the regulation of ERα deubiquitination and stabilization. MINDY1 interacted with ERα protein and enhanced its stability via removing the K48-linked ubiquitin chain from ERα. Furthermore, our data demonstrated that MINDY1 may promote breast cancer progression through the expression of ERα. Therefore, MINDY1 could be a potential therapeutic target for ERα-positive breast cancer.

## Supplementary information

Supplementary legend

Figure S1

Figure S2

Figure S3

## References

[1] Bray F, Ferlay J, Soerjomataram I, Siegel RL, Torre LA, Jemal A (2018). Global cancer statistics 2018: GLOBOCAN estimates of incidence and mortality worldwide for 36 cancers in 185 countries. CA Cancer J Clin.

[2] Maughan KL, Lutterbie MA, Ham PS (2010). Treatment of breast cancer. Am Fam Physician.

[3] Sledge GW, Mamounas EP, Hortobagyi GN, Burstein HJ, Goodwin PJ, Wolff AC (2014). Past, present, and future challenges in breast cancer treatment. J Clin Oncol.

[4] Diaby V, Tawk R, Sanogo V, Xiao H, Montero AJ (2015). A review of systematic reviews of the cost-effectiveness of hormone therapy, chemotherapy, and targeted therapy for breast cancer. Breast Cancer Res Treat.

[5] Onitilo AA, Engel JM, Greenlee RT, Mukesh BN (2009). Breast cancer subtypes based on ER/PR and Her2 expression: comparison of clinicopathologic features and survival. Clin Med Res.

[6] Dowsett M, Goldhirsch A, Hayes DF, Senn HJ, Wood W, Viale G (2007). International web-based consultation on priorities for translational breast cancer research. Breast Cancer Res.

[7] Ng HW, Perkins R, Tong W, Hong H (2014). Versatility or promiscuity: the estrogen receptors, control of ligand selectivity and an update on subtype selective ligands. Int J Environ Res Public Health.

[8] Greene GL, Press MF (1986). Structure and dynamics of the estrogen receptor. J Steroid Biochem.

[9] Miyoshi Y, Murase K, Saito M, Imamura M, Oh K (2010). Mechanisms of estrogen receptor-α upregulation in breast cancers. Med Mol Morphol.

[10] Tecalco-Cruz AC, Ramírez-Jarquín JO (2017). Mechanisms that increase stability of estrogen receptor alpha in breast cancer. Clin Breast Cancer.

[11] Cariou S, Donovan JC, Flanagan WM, Milic A, Bhattacharya N, Slingerland JM (2000). Down-regulation of p21WAF1/CIP1 or p27Kip1 abrogates antiestrogen-mediated cell cycle arrest in human breast cancer cells. Proc Natl Acad Sci USA.

[12] Giulianelli S, Vaqué JP, Wargon V, Soldati R, Vanzulli SI, Martins R (2012). [The role of estrogen receptor alpha in breast cancer cell proliferation mediated by progestins]. Med (B Aires).

[13] Cardoso F, Costa A, Senkus E, Aapro M, André F, Barrios CH (2017). 3rd ESO-ESMO International Consensus Guidelines for Advanced Breast Cancer (ABC 3). Ann Oncol.

[14] Li X, Lu J, Zhang L, Luo Y, Zhao Z, Li M (2020). Clinical implications of monitoring ESR1 mutations by circulating tumor DNA in estrogen receptor positive metastatic breast cancer: a pilot study. Transl Oncol.

[15] Reinhardt F, Franken A, Meier-Stiegen F, Driemel C, Stoecklein NH, Fischer JC (2019). Diagnostic leukapheresis enables reliable transcriptomic profiling of single circulating tumor cells to characterize inter-cellular heterogeneity in terms of endocrine resistance. Cancers (Basel).

[16] Zhuang T, Yu S, Zhang L, Yang H, Li X, Hou Y (2017). SHARPIN stabilizes estrogen receptor α and promotes breast cancer cell proliferation. Oncotarget.

[17] Fan M, Park A, Nephew KP (2005). CHIP (carboxyl terminus of Hsc70-interacting protein) promotes basal and geldanamycin-induced degradation of estrogen receptor-alpha. Mol Endocrinol.

[18] Sun J, Zhou W, Kaliappan K, Nawaz Z, Slingerland JM (2012). ERα phosphorylation at Y537 by Src triggers E6-AP-ERα binding, ERα ubiquitylation, promoter occupancy, and target gene expression. Mol Endocrinol.

[19] Eakin CM, Maccoss MJ, Finney GL, Klevit RE (2007). Estrogen receptor alpha is a putative substrate for the BRCA1 ubiquitin ligase. Proc Natl Acad Sci USA.

[20] Hashizume R, Fukuda M, Maeda I, Nishikawa H, Oyake D, Yabuki Y (2001). The RING heterodimer BRCA1-BARD1 is a ubiquitin ligase inactivated by a breast cancer-derived mutation. J Biol Chem.

[21] Bhatt S, Xiao Z, Meng Z, Katzenellenbogen BS (2012). Phosphorylation by p38 mitogen-activated protein kinase promotes estrogen receptor α turnover and functional activity via the SCF(Skp2) proteasomal complex. Mol Cell Biol.

[22] Saji S, Okumura N, Eguchi H, Nakashima S, Suzuki A, Toi M (2001). MDM2 enhances the function of estrogen receptor alpha in human breast cancer cells. Biochem Biophys Res Commun.

[23] Xia X, Liao Y, Huang C, Liu Y, He J, Shao Z (2019). Deubiquitination and stabilization of estrogen receptor α by ubiquitin-specific protease 7 promotes breast tumorigenesis. Cancer Lett.

[24] Tang J, Luo Y, Tian Z, Liao X, Cui Q, Yang Q (2020). TRIM11 promotes breast cancer cell proliferation by stabilizing estrogen receptor α. Neoplasia.

[25] Tang J, Wu Z, Tian Z, Chen W, Wu G (2021). OTUD7B stabilizes estrogen receptor α and promotes breast cancer cell proliferation. Cell Death Dis.

[26] Tian Z, Tang J, Liao X, Gong Y, Yang Q, Wu Y (2020). TRIM8 inhibits breast cancer proliferation by regulating estrogen signaling. Am J Cancer Res.

[27] Walter P, Green S, Greene G, Krust A, Bornert JM, Jeltsch JM (1985). Cloning of the human estrogen receptor cDNA. Proc Natl Acad Sci USA.

[28] Shao W, Brown M (2004). Advances in estrogen receptor biology: prospects for improvements in targeted breast cancer therapy. Breast cancer Res.

[29] Portman N, Milioli HH, Alexandrou S, Coulson R, Yong A, Fernandez KJ (2020). MDM2 inhibition in combination with endocrine therapy and CDK4/6 inhibition for the treatment of ER-positive breast cancer. Breast Cancer Res.

[30] Turner N, Pearson A, Sharpe R, Lambros M, Geyer F, Lopez-Garcia MA (2010). FGFR1 amplification drives endocrine therapy resistance and is a therapeutic target in breast cancer. Cancer Res.

[31] Kuang Y, Siddiqui B, Hu J, Pun M, Cornwell M, Buchwalter G (2018). Unraveling the clinicopathological features driving the emergence of ESR1 mutations in metastatic breast cancer. NPJ Breast Cancer.

[32] Wang C, Fu M, Angeletti RH, Siconolfi-Baez L, Reutens AT, Albanese C (2001). Direct acetylation of the estrogen receptor alpha hinge region by p300 regulates transactivation and hormone sensitivity. J Biol Chem.

[33] Anbalagan M, Rowan BG (2015). Estrogen receptor alpha phosphorylation and its functional impact in human breast cancer. Mol Cell Endocrinol.

[34] Tharun IM, Nieto L, Haase C, Scheepstra M, Balk M, Möcklinghoff S (2015). Subtype-specific modulation of estrogen receptor-coactivator interaction by phosphorylation. ACS Chem Biol.

[35] Fu L, Cui CP, Zhang X, Zhang L (2020). The functions and regulation of Smurfs in cancers. Semin cancer Biol.

[36] Sharma B, Bhatt TK (2017). Genome-wide identification and expression analysis of E2 ubiquitin-conjugating enzymes in tomato. Sci Rep.

[37] Liao TL, Wu CY, Su WC, Jeng KS, Lai MM (2010). Ubiquitination and deubiquitination of NP protein regulates influenza A virus RNA replication. EMBO J.

[38] Zhang X, Kuramitsu Y, Ma A, Zhang H, Nakamura K (2016). Endoplasmic reticulium protein profiling of heat-stressed Jurkat cells by one dimensional electrophoresis and liquid chromatography tandem mass spectrometry. Cytotechnology.

[39] Wang B, Xie M, Li R, Owonikoko TK, Ramalingam SS, Khuri FR (2014). Role of Ku70 in deubiquitination of Mcl-1 and suppression of apoptosis. Cell Death Differ.

[40] Song H, Tao L, Chen C, Pan L, Hao J, Ni Y (2015). USP17-mediated deubiquitination and stabilization of HDAC2 in cigarette smoke extract-induced inflammation. Int J Clin Exp Pathol.

[41] Abdul Rehman SA, Kristariyanto YA, Choi SY, Nkosi PJ, Weidlich S, Labib K (2016). MINDY-1 Is a Member of an Evolutionarily Conserved and Structurally Distinct New Family of Deubiquitinating Enzymes. Mol Cell.

