# Correction to: MINDY1 promotes breast cancer cell proliferation by stabilizing estrogen receptor α

**DOI:** 10.1038/s41419-022-04549-7

**Published:** 2022-01-27

**Authors:** Jianing Tang, Yongwen Luo, Guo Long, Ledu Zhou

**Affiliations:** 1 Department of Liver Surgery, Xiangya Hospital, Central South University, Changsha, China; 2 Department of Urology, Zhongnan Hospital of Wuhan University, Wuhan, China

**Keywords:** Cancer, Ubiquitylation

Correction to: *Cell Death and Disease* 10.1038/s41419-021-04244-z, published online 13 October 2021

The original version of this article unfortunately contained a mistake. There was an error in Fig. 7. The authors apologize for the mistake. The original article has been corrected.

