# Supplementary material for: MINDY1 promotes breast cancer cell proliferation by stabilizing estrogen receptor α
Source: Cell Death Dis. 2021 Oct 13;12(10):937. doi: 10.1038/s41419-021-04244-z (PMC8514509; doi:10.1038/s41419-021-04244-z)
Supplement: Supplementary file 1 — Supplementary legend [file 41419_2021_4244_MOESM1_ESM.docx]

**Supplementary legend**

**Figure 1. MINDY1 regulates ERα signaling activity in breast cancer cells..** (A). The siRNAs specific to each deubiquitinating enzyme (MINDY1-4) were transfected into MCF-7 cells. After 48 h, cells were lysed and the ERα protein level was analyzed by Western blot. Relative ERα protein level was normalized to GAPDH. (B). MINDY1 depletion did not affect ERα mRNA level. (C). MINDY1 depletion effect on ERα protein level. MCF-7 cells were transfected with siMINDY1 or siControl. After 48 h, cells were treated with either ethanol or 10 nM estradiol for 6 h. ERα protein levels were determined by the western blot analysis. (D). MINDY1 enhanced ERE-luciferase activity. Breast cancer cells were transfected with MINDY1 or Vector together with ERE luciferase reporter plasmid. Cells were treated with 10 nM estradiol or vehicle. Luciferase activity was measured 48 h after transfection. The experiment was independently repeated three times with three replicates.

*, *P value < 0.05; **, P value < 0.01; ***, P value < 0.001.*

**Figure S2.** Wild-type MINDY1, but not MINDY1^C137A^ possesses DUB activity towards polyubiquitinated ERα *in vitro.* Ubiquitinated ERα was purified from HEK293 cells transfected with HA-Ub, and Flag- ERα plasmids using anti-Flag affinity purification method. The ubiquitinated ERα was incubated with GST-tagged wildtype MINDY1, and MINDY1^C137A^ proteins purified from bacteria in 20 µl reaction buffer containing at 37 °C for 2 h. The reactions were stopped by boiling in 1X SDS sample buffer and analyzed using IB with anti-HA and anti-GST antibodies.

**Figure S3. MINDY1 knockdown enhances the sensitivity of ERα+ breast cancer cells to tamoxifen.** (A). MINDY1 depletion decreased cell proliferation in both vehicle and estradiol treated conditions (B). Cells were exposed to MINDY1 siRNA and tamoxifen (selleck, 20 μM/L). Cell viability was detected. (C). Cells were exposed to MINDY1 siRNA and tamoxifen. Cell apoptosis was detected by flow cytometry.
