# Supplementary figures and images for: MINDY1 promotes breast cancer cell proliferation by stabilizing estrogen receptor α
Source: Cell Death Dis. 2021 Oct 13;12(10):937. doi: 10.1038/s41419-021-04244-z (PMC8514509; doi:10.1038/s41419-021-04244-z)

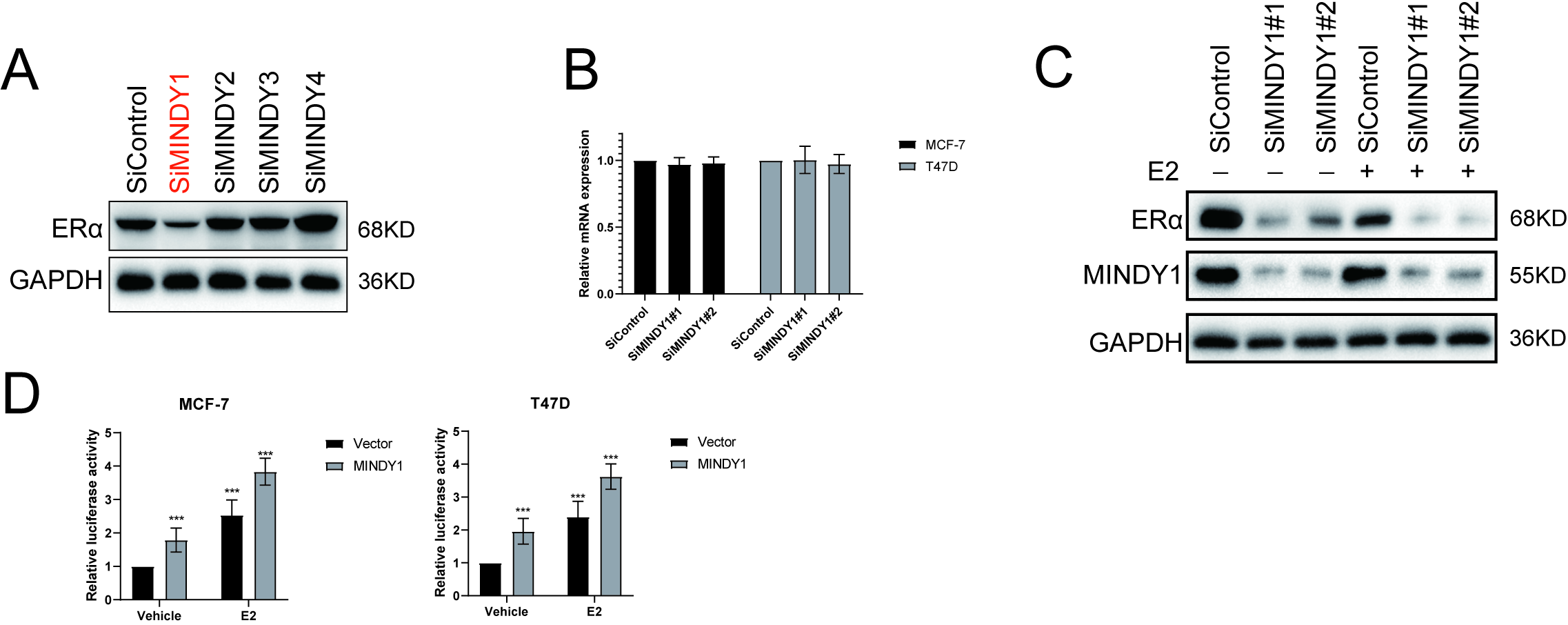

Supplement: Supplementary file 2 — Figure S1 [file 41419_2021_4244_MOESM2_ESM.tif]

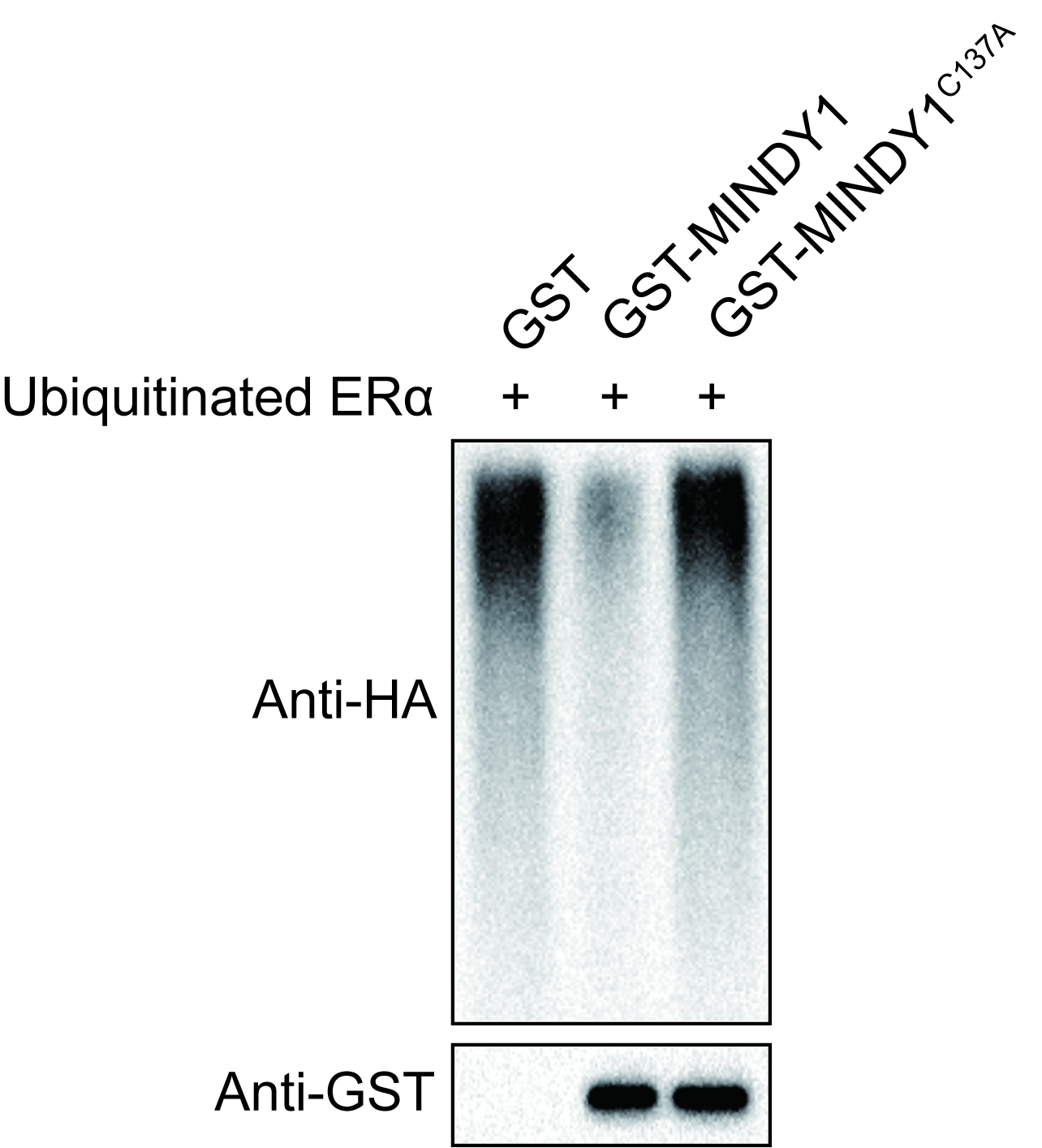

Supplement: Supplementary file 3 — Figure S2 [file 41419_2021_4244_MOESM3_ESM.tif]

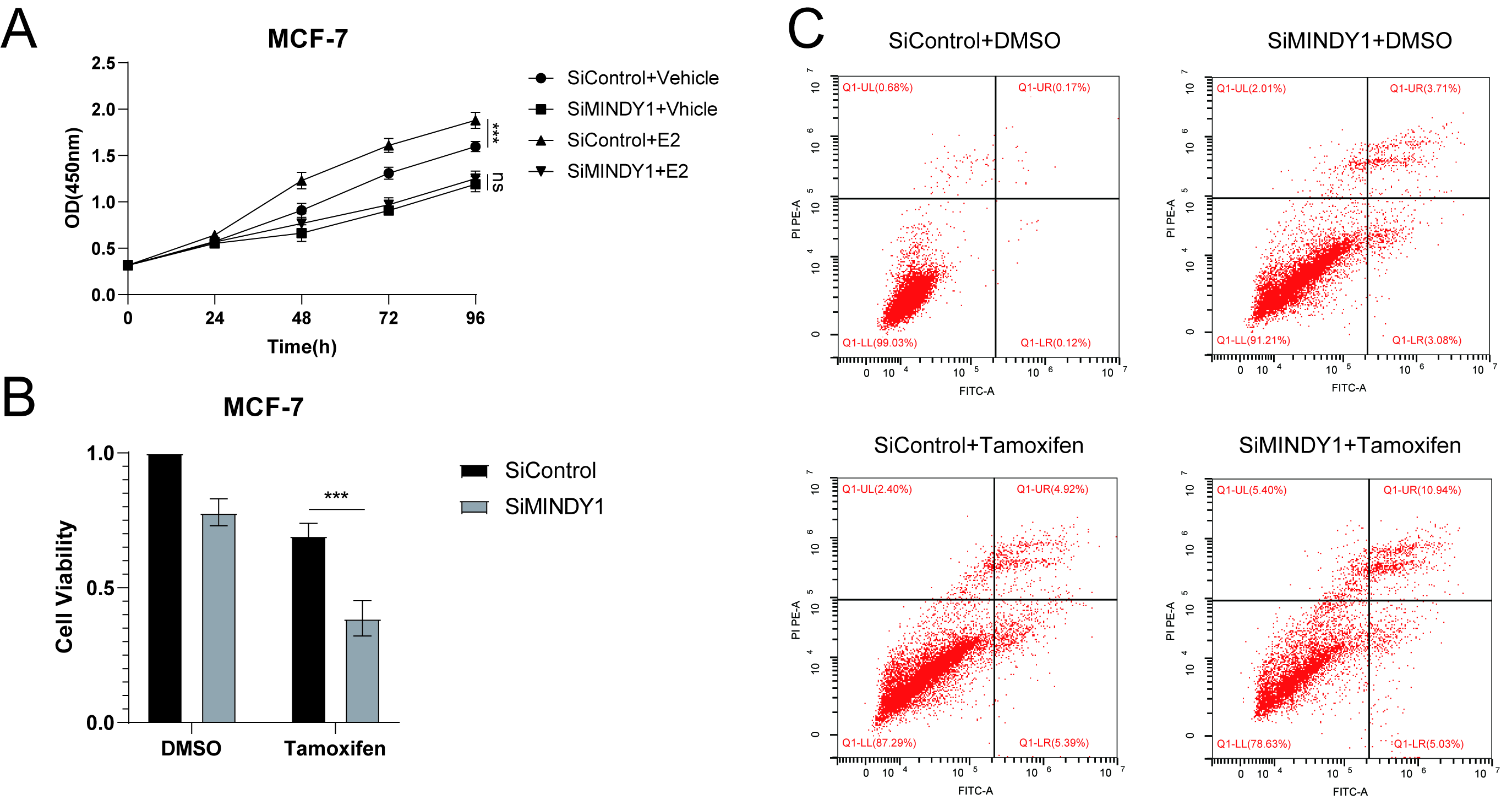

Supplement: Supplementary file 4 — Figure S3 [file 41419_2021_4244_MOESM4_ESM.tif]
